# To Reduce Weight

**Published:** 1887-06

**Authors:** 


TO REDUCE WEIGHT.—Eat, to the extent of satisfying a natural appetite, of lean meat, poultry, game, eggs, milk moderately, green vegetables, turnips, succulent fruits, tea or coffee. Drink limejuice, lemonade, and acid drinks. Avoid fat, butter, cream, sugar, pastry, rice, sago, tapioca, corn-starch, potatoes, carrots, beets, parsnips, and sweet wines. Exercise freely.—*Ibid.*

